# Genome-wide expression reveals potential biomarkers in breast cancer bone metastasis

**DOI:** 10.1515/jib-2021-0041

**Published:** 2022-04-08

**Authors:** Yashbir Singh, Naidu Subbarao, Abhinav Jaimini, Quincy A. Hathaway, Amina Kunovac, Bradley Erickson, Vishnu Swarup, Himanshu Narayan Singh

**Affiliations:** Department of Radiology, Mayo Clinic, Rochester, MN, USA; School of Computational and Integrative Sciences, Jawaharlal Nehru University, New Delhi, India; Divisions of PET Imaging, MIRC, Institute of Nuclear Medicine and Allied Sciences (INMAS), Timarpur, Delhi, India; Department of Cardiology, West Virginia University School of Medicine, Heart & Vascular Institute, Morgantown, WV, USA; Division of Exercise Physiology, West Virginia University School of Medicine, Morgantown, WV, USA; Department of Neurology, All India Institute of Medical Sciences, New Delhi, India; Aix-Marseille University, INSERM, TAGC, UMR 1090, Marseille 13288, France; MTA Infotech, Varanasi, India

**Keywords:** biological networking, biomarkers, breast cancer, drug targets, genomics

## Abstract

Breast cancer metastases are most commonly found in bone, an indication of poor prognosis. Pathway-based biomarkers identification may help elucidate the cellular signature of breast cancer metastasis in bone, further characterizing the etiology and promoting new therapeutic approaches. We extracted gene expression profiles from mouse macrophages from the GEO dataset, GSE152795 using the GEO2R webtool. The differentially expressed genes (DEGs) were filtered by log2 fold-change with threshold 1.5 (FDR < 0.05). STRING database and Enrichr were used for GO-term analysis, miRNA and TF analysis associated with DEGs. Autodock Vienna was exploited to investigate interaction of anti-cancer drugs, Actinomycin-D and Adriamycin. Sensitivity and specificity of DEGs was assessed using receiver operating characteristic (ROC) analyses. A total of 61 DEGs, included 27 down-regulated and 34 up-regulated, were found to be significant in breast cancer bone metastasis. Major DEGs were associated with lipid metabolism and immunological response of tumor tissue. Crucial DEGs, Bcl3, ADGRG7, FABP4, VCAN, and IRF4 were regulated by miRNAs, miR-497, miR-574, miR-138 and TFs, CCDN1, STAT6, IRF8. Docking analysis showed that these genes possessed strong binding with the drugs. ROC analysis demonstrated Bcl3 is specific to metastasis. DEGs Bcl3, ADGRG7, FABP4, IRF4, their regulating miRNAs and TFs have strong impact on proliferation and metastasis of breast cancer in bone tissues. In conclusion, present study revealed that DEGs are directly involved in of breast tumor metastasis in bone tissues. Identified genes, miRNAs, and TFs can be possible drug targets that may be used for the therapeutics. However, further experimental validation is necessary.

## 1 Introduction

Worldwide, breast cancer is known to contribute to the highest incidence of cancer-related mortality in women [1]. Metastatic recurrence in breast cancer is associated with poor prognosis and is a leading cause of cancer related death in women worldwide [2]. Despite improved treatments and surgical interventions, about 30% of patients lose their life due to metastasis [3].

Among metastasis in several other tissues and organs in the body (lungs, liver and brain, in addition to lymph nodes), bone is one of the most common sites of invasion in breast cancer [1, 4]. Metastasis to bone typically results in a poor prognosis, reducing life expectancy to 2–3 years post-diagnosis. The tumor cell mass exerts mechanical pressure that can contribute to bone pain. Pain may also occur due to release of inflammatory cytokines from the tumor cells themselves or by altering the bone microenvironment and bone homeostasis in adjacent areas [5].

Bone cancer metastases usually occur in the axial skeleton, in areas with active hematopoiesis and high red marrow content. The microenvironment is fundamental in attracting tumor cells to the bone as well as promoting tumor progression. The composition of the tumor microenvironment changes during tumor development, evolving to meet the demands of the growing neoplasm [6]. These differences affect the progression of cancer and how it responds to therapeutic agents and their ultimate efficacy. An elaborate network of signaling pathways orchestrates the communication between cancer cells and the surrounding stroma. Chemotherapy, radiation therapy, and surgical removal of the tumors are standard methods implemented to cure breast cancers. The usage of thermoset based polymers [7–9] as a drug delivery system is currently a common approach when exploring chemotherapy as an option [10]. Several chemotherapeutic molecules have proven their potential in treatment of breast tumour. Actinomycin D and its analogues and Adriamycin (aka doxorubicin) have been shown to reduce the migration and invasion of tumour cells [11, 12].

In this study, we explore different signaling pathways and associated molecular mechanisms of underlying primary breast cancer development, along with the formation of metastases in the bone. The study will also illustrate biomarkers associated with disease pathophysiology and highlight potential new therapeutics.

## 2 Materials

The expression profile information of mouse macrophages was extracted from the GEO dataset, GSE152795 (https://www.ncbi.nlm.nih.gov/geo/query/acc.cgi?acc=GSE152795). The annotation was performed using Affymetrix GeneChip^®^ Mouse Gene 2.0ST Array. The dataset included breast cancer bone metastasis in healthy mouse strains, with a sample size of five in each group. Bone metastasis was generated through intra-cardiac injection of 1 × 10^5^ tumor cells into 4-week-old female mice, while healthy mice were not subjected to intra-cardiac injection.

## 3 Methods

### 3.1 Identification of differential expressed genes (DEGs)

The GEO2R (http://www.ncbi.nlm.nih.gov/geo/geo2r/) web tool was used to compare mRNA expression between breast cancer metastasis and normal tissue samples in mice. The Benjamini and Hochberg false discovery rate (FDR) method was used to filter out false-positive results, and the adjusted *p* values were calculated. The differentially expressed genes were filtered based on log2 fold change, with a threshold value ≥1 and *p* < 0.05. To determine the difference among samples and graphically depict the data, we used a built-in tool of GEO2R Uniform Manifold Approximation and Projection (UMAP). A volcano plot showing differentially expressed genes was constructed using the R Language ggplot package.

The receiver operating characteristics (ROC) curve was plotted to determine the specificity and sensitivity of the identified DEGs towards controls and tumor samples using Stata v17.0. The statistical significance level was established as *p* value < 0.05.

### 3.2 Network analysis

The protein network analysis of the differentially expressed genes was performed using the STRING database (http://string-db.org), which provides a physical and functional association of proteins. It also provides information about how genes are enriched via various pathways. The functional and pathway enrichment analyses were performed with the cut-off criterion *p*-value < 0.05. Protein-protein interaction visualization was done through the STRING database.

### 3.3 Identification of transcription factors and miRNAs binding to DEGs

The Enrichr webtool [13] was exploited to extract miRNA targeting the identified genes. Significantly (*p* < 0.05) enriched miRNAs were extracted. The information on protein-protein interactions (PPI) of TFs was also fetched from the Enrichr webtool.

### 3.4 Molecular docking

#### 3.4.1 Protein preparation

The 3D structure of the targets FABP4 (PDB ID: 3P6D) and IRF4 (PDB ID: 7JM4) were extracted from the Protein Databank. The protein structure of ADGRG7 was extracted from the AlphaFold Protein Structure Database (https://alphafold.ebi.ac.uk/entry/Q96K78). Local minimization of the structure was done by the GROMOS96 (43B1 parameter set) and the Swiss-pdbViewer used for implementation [14]. The CASTp (Computed Atlas of Surface Topography of proteins) web tool was used to extract ligand binding site information [15].

#### 3.4.2 Preparation of ligand molecules

The three-dimensional structure of the drugs, Actinomycin D (PubChem ID: 31703) and Adriamycin (PubChem ID: 457193), were extracted from the PubChem database [16]. The ligand structures were converted to pdbqt format by OpenBabel software [17].

#### 3.4.3 Binding in silico

The protein preparation in pdbqt-format was executed using AutodockTools 1.5.4 suite [18]. The molecular docking analysis was performed to develop an improved modeled structure by Autodock Vina Software [19]. After docking analysis, the obtained structures were detected by the Protein-Ligand Interaction Profiler (PLIP) web server (Technical University of Dresden) and tabularized for further analysis [20].

**Figure 1:**
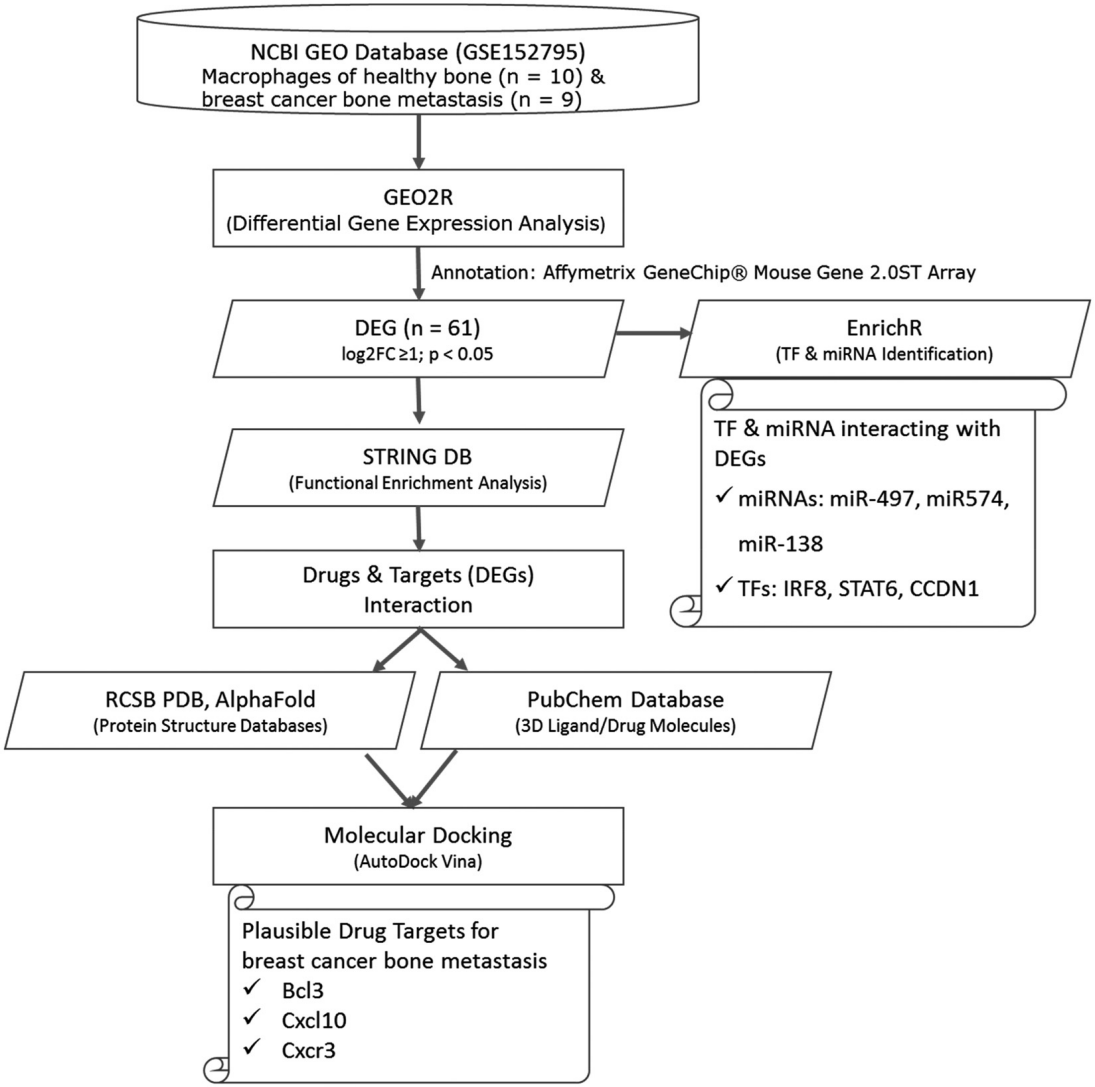
The overall workflow of the methods employed in identifying drug targets, TFs and miRNAs.

Figure 1 presents an overview of methods used in present differential expression analysis of macrophages of healthy bone & breast cancer bone metastasis.

## 4 Results

### 4.1 Sample relatedness

**Figure 2:**
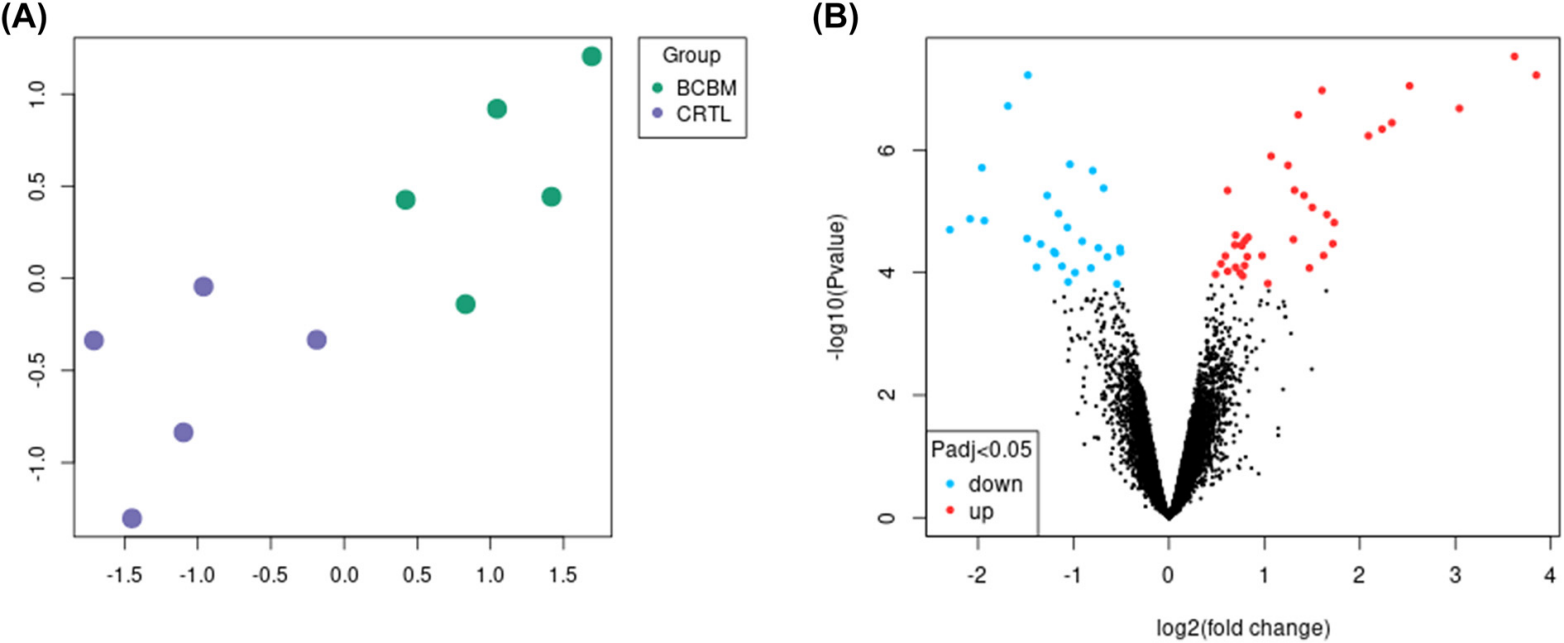
Identification of differentially expressed genes. (A) Uniform manifold approximation and projection (UMAP) plot shows sample relatedness. Healthy bone samples are shown in blue-grey and cancer samples are shown in green. (B) Volcano plot is showing differentially expressed genes. Red color: upregulated genes (logFC > 1 and padj < 0.05). Blue shows: downregulated genes (logFC < −1 and padj < 0.05). Gray color denotes no significant change in the expression.

The sample and group relationships were identified using the Uniform Manifold Approximation and Projection (UMAP) dimension reduction technique; this provided clustering of the tumor and normal tissues into their respective groups. By color-coding of the sample group on the UMAP, we observed a clear difference between the cancer metastasis group and that of the controls, as shown in Figure 2A, where green depicts the breast cancer bone metastasis samples, and healthy samples are shown in blue-grey.

### 4.2 Differentially expressed genes

A total of 61 genes were found to be significantly differentially expressed in breast cancer bone metastasis. This included 27 down-regulated genes shown in blue, and 34 up-regulated genes depicted in red (Figure 2B, Supplementary Table S1). Two genes found to be up-regulated by greater than a factor of 3 were Saa3 (Serum amyloid A-3 protein) and Lipg (Supplementary Table S1). They were found to be directly correlated with breast cancer. A new gene, LIPG, was recently found to express and play a role in breast cancer proliferation, tumorigenicity, and metastasis [21]. An acute-phase protein produced by hepatocytes and adipose tissues named Serum amyloid A (SAA) binds to pattern recognition receptors (PRRs), and has a potential role in tumor promotion [22].

### 4.3 Functional enrichment analysis

**Figure 3:**
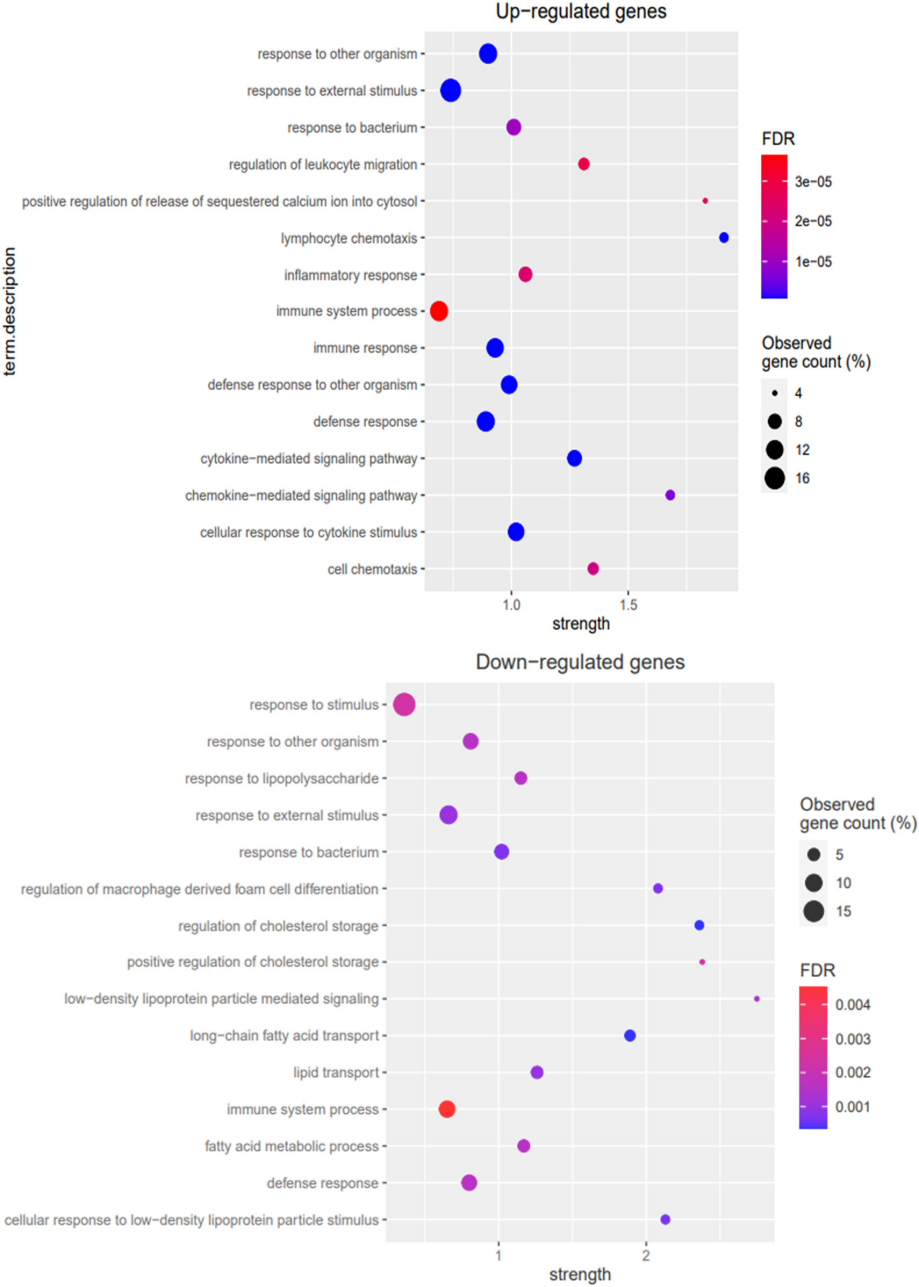
Bubble plot of the GO analysis of DEGs. The top 20 items of ’biological process’ in upregulated genes (upper panel) and downregulated genes (lower panel) were displayed with the parameters strength and −log10 *p*-value. Strength: Log10 (observed/expected). This measure describes how large the enrichment effect is. It is the ratio between (i) the number of proteins in your network that are annotated with a term and (ii) the number of proteins that we expect to be annotated with this term in a random network of the same size.

Our results demonstrate that a total of 122 and 105 GO-terms were found to be significantly (FDR < 0.05) enriched for up-regulated genes and down-regulated genes, respectively (Supplementary Tables S2, S3). The top 20 biological processes highly enriched differentially expressed genes were found to be associated with immunological activation and lipid metabolism (Figure 3). The differentially expressed genes were found to be significantly associated with receptor activity and/or binding functions. The differentially expressed genes were major components of the cell membrane or extracellular membrane. Out of 34 up-regulated genes, only 19 genes were found to be associated with the top 20 enriched biological processes that were related to immunological responses. However, 21 down-regulated genes were enriched in the top 20 biological processes associated with lipid metabolism and immunological responses (Supplementary Table S2, S3).

### 4.4 TFs and miRNAs binding to DEGs

**Figure 4:**
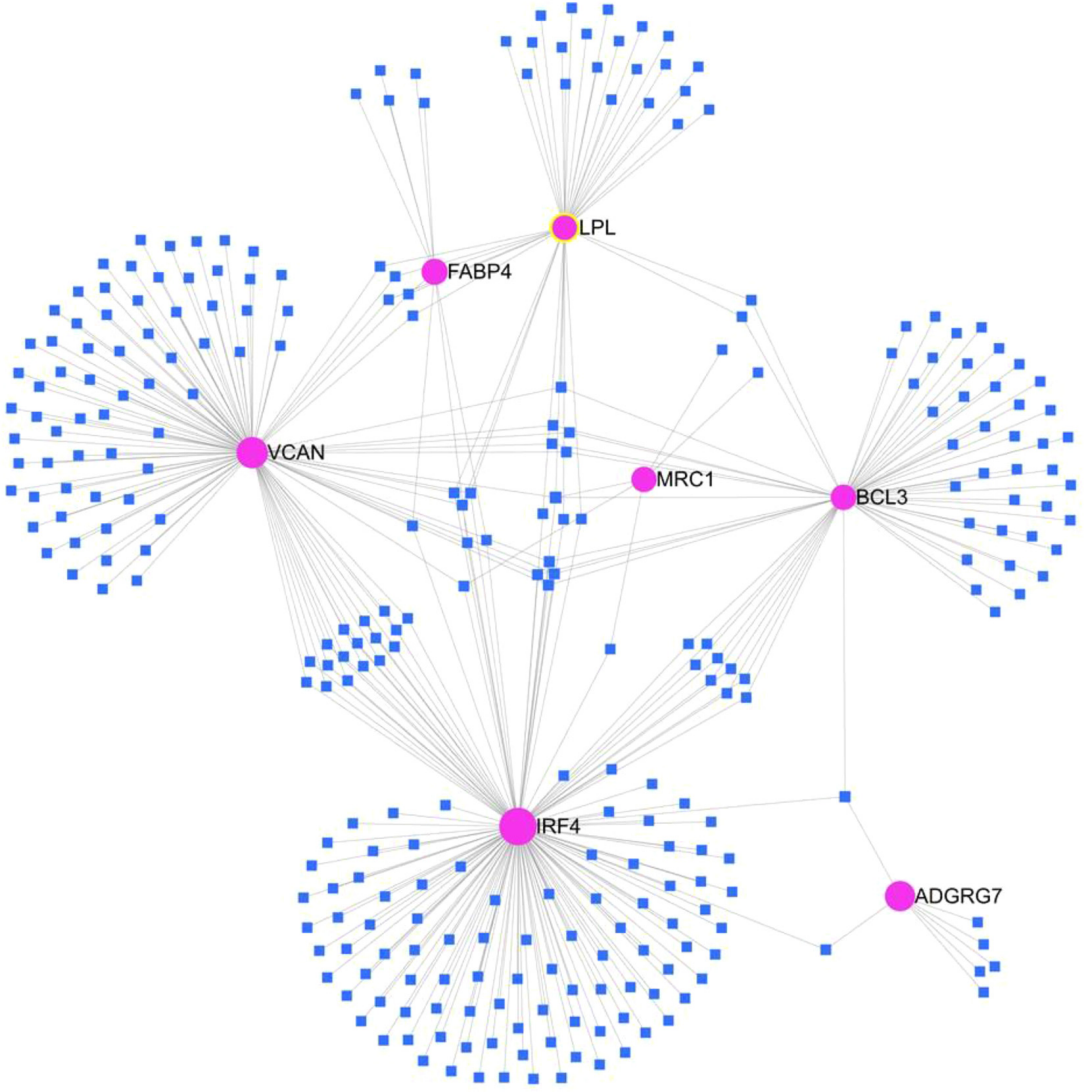
Network of identified miRNAs and their target genes made by miRNet 2.0. The round pink spots represent DEGs and the blue coloured squares represent the miRNAs.

The Enrichr identified 28 miRNAs (Supplementary Table S4) which are targeting DEGs. The miRNA-mRNA interaction map, made by using miRNet v2.0 showed seven of these DEGs having strong interactions with miRNAs (Figure 4). Genes IRF4 and VCAN showed the highest degree of interactions while MRC1 showed the least level of interaction.

**Figure 5:**
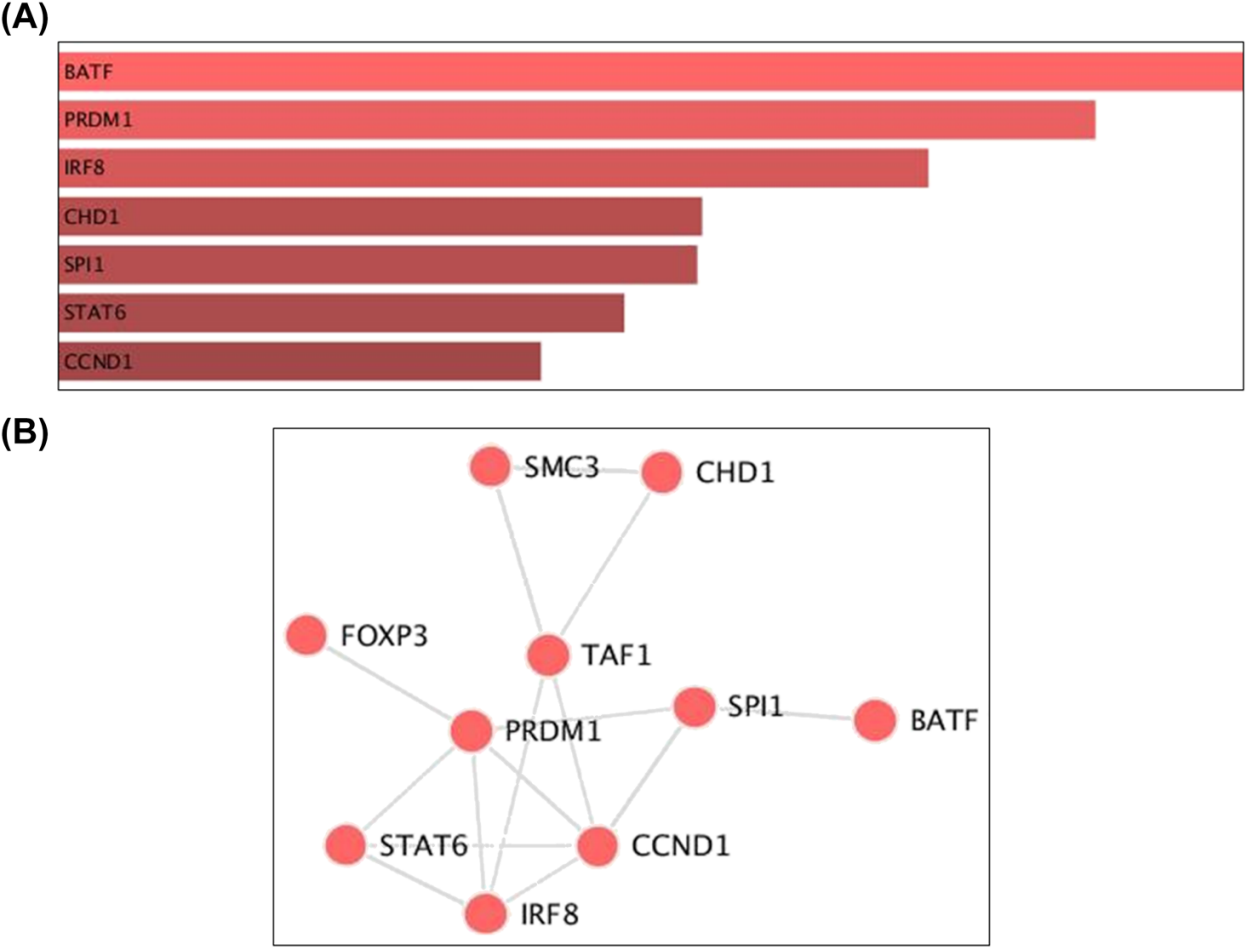
Identified significant TFs targeting DEGs and their interaction with other TFs/proteins.

Seven significant TFs (p < 0.05) were identified by Enrichr (Figure 5A, Supplementary Table S5). The interactions of these TFs with other proteins/TFs in Figure 5B clearly show strong interaction. The interaction between TFs showed PRDM1 connected with various other TFs which depicted its central role in pathogenesis (Figure 5B).

### 4.5 ROC analysis

**Figure 6:**
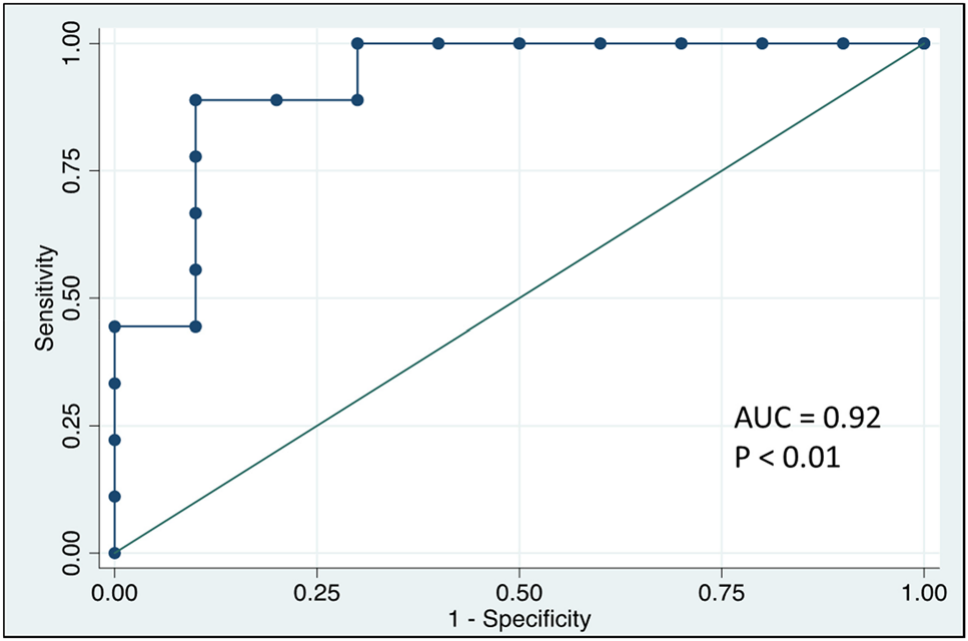
ROC curve analysis to test the validity of Bcl3 gene expression in discriminating samples of patients and controls.

In the Bcl3 gene, the fitted ROC curve is estimated with the assumption of binomial distribution (Figure 6). The parametric estimate of the area under the fitted ROC curve was 0.92 and its 95% confidence interval are 0.73972 and 0.99867. Only the Bcl3 gene showed significant relevance with sensitivity and specificity with the disease as compared to other genes (Supplementary Figure S1A–H).

### 4.6 Interaction of DEGs and most common drugs used in treatment

**Figure 7:**
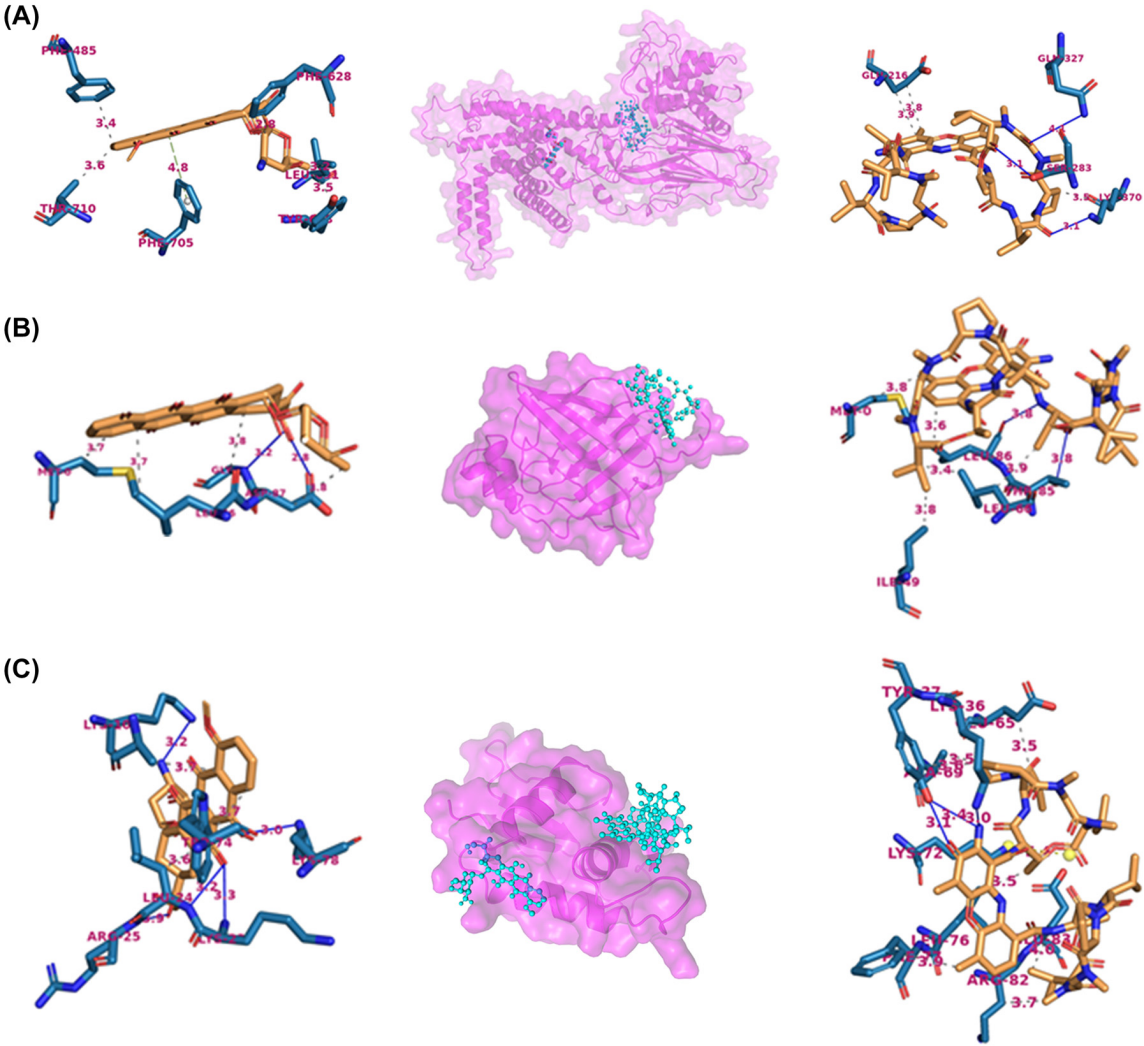
The interactions that established after docking the drugs, adriamycin (left of the protein drug complex) and actinomycin (right of the protein drug complex) against the three target proteins: (A) ADGRG7, (B) FABP4 and (C) IRF4. Protein is represented in ribbon (magenta color) and ligands are shown in ball stick model. Hydrophobic Interaction are shown by dashed lines and Hydrogen Bond are shown by blue densed lines.

The binding site for the target proteins ADGRG7, FABP4 and IRF4 were predicted to be located around ILE (Chain A; Sequence ID: 34), PHE (Chain A; Sequence ID: 16) and GLY (Chain G; Sequence ID: 22), respectively (Figure 7 A–C, Supplementary Table S7–S9). The molecular docking interaction analysis of Actinomycin and Adriamycin drugs, with all the protein targets showed high binding energy (Table 1).

**Table 1:** Binding site information and docking analysis of drugs with the protein targets.

Protein target	Binding site			Free binding energy (kcal/mol)	
	Chain	Seq ID	Amino acid	Actinomycin	Adriamycin
ADGRG7	A	24	ILE	−9.4	−8.1
FABP4	A	16	PHE	−8.5	−7.9
IRF4	G	22	GLY	−8.0	−7.6

The free binding energies of the molecules were found to be ranged from −7.6 to −9.4 kcal/mol. The protein-ligand complex of ADGRG7 with both drugs was observed with the highest binding energy (ADGRG7-Actinomycin complex ∆*G* = −9.4 kcal/mol; ADGRG7-Adriamycin complex ∆G = −8.1 kcal/mol). The free binding energy of ADGRG7-Actinomycin complex was highest (−)9.4 kcal/mol and the IRF4-Adriamycin complex showed lowest binding energy of (−)7.6 kcal/mol.

## 5 Discussion

The most common site for breast cancer metastases, bone, is involved in about 70% of all metastatic patients [23, 24]. Among breast cancer patients, approximately 14% of patients develop bone metastasis at 15 years post-follow-up [25]. Tumor cells regulate several factors in the bone microenvironment for their maintenance and growth. By releasing cytokines, chemokines, and growth factors, the bone microenvironment inhibits colonization of cancer cells in healthy bones, or supports colonization in cancerous conditions [26, 27]. Production of interferon-γ (IFN-γ) by activated CD4 + T cells inhibits osteoclasts’ activity, thereby protecting bone [28]. Whereas after tumor cell invasion, osteoclastogenesis is promoted by the release of osteoprotegerin (OPG) and cytokines (IL6, IL15 etc.), leading to bone destruction [29]. Thus, an altered balance within the immune system between pro-tumorigenic and anti-tumorigenic molecules decides the fate of tumor cells in bone.

In addition, immune cell plasticity has extensive implications in the pathogenesis and resolution of metabolic disorders: cancers, autoimmune diseases, and chronic inflammatory disorders. In metabolic disorders such as obesity, immune cells interact with various classes of lipids, which can control the plasticity of macrophages and T-lymphocytes. Alterations in lipid metabolism are directly associated with immune cell activation and are also involved in mechanisms associated with several human diseases such as cancer [30]. The proliferation of tumor cells and metastases requires a large amount of energy which is obtained by alterations in lipid metabolism [31]. Several clinical studies have demonstrated high levels of lipids (triglycerides, high density- and low density lipoproteins) in blood plasma of breast cancer patients [32]. Moreover, accumulation of these molecules was correlated with a high propensity of metastases in breast cancer patients. Elevated free fatty acid levels also inhibit fibrin degradation, facilitating breast cancer invasion and metastasis [33]. Thus, a dysregulated immune system and lipid metabolism promote metastases in breast cancer.

This study demonstrates that the up-regulated genes in breast cancer metastases are found to be associated with immune responses. On the other hand, down-regulated genes were involved with the immune response as well as lipid metabolism biological processes (Figure 3).

Four-fold down-regulation of the platelet glycoprotein *Cd36* was observed in mouse macrophages of breast cancer bone metastases samples (Table S1). The Cd36 down-regulation was also observed in primary tumor epithelium, and its possible association with breast cancer metastases was reported [34, 35]. Peroxisome proliferator-activated receptor-gamma (*PPAR-γ*) is a nuclear hormone receptor superfamily transcription factor involved in metabolic functions as well as a suppressor of breast cancer tumors [36] and was estimated to be down-regulated two-fold in tumors. Similarly, other downregulated genes (*Fabp4, Mrc1, Lpl, Cd209d, and Cd209a*) were also found to be involved in breast cancer and may be associated with bone metastases. However, further investigation is required to confirm their association with bone metastases. Lipoprotein lipase (Lpl) was observed to be depleted in the disease condition (Table S1). Lpl functions as a triglyceride hydrolase and receptor-mediated factor for lipoprotein uptake [37]. Loss of Lpl expression was reported to be directly associated with lipid metabolism and to cause hyperlipidemia [38, 39]. A previous study suggests that down-regulation of Lpl may be the result of epigenetic alterations, such as CpG island hypermethylation and/or histone modification [40]. Altered genomic DNA is also reported to contribute to the loss of Lpl. The cancer susceptibility gene deleted in breast cancer (Dcb2), known as Rho-related BTB domain containing 2 (Rohbtb2), mapped closed to the Lpl gene which may also be affected by Lpl deletion and could therefore exert a combined impact on promoting carcinogenesis [40, 41]. Lpl is found to suppress TNFa and IFNg associated inflammation activating genes through inactivation of transcription factor NF-kB [40, 42]. Therefore, loss of Lpl activity may promote carcinogenesis.

Several significantly up-regulated genes were identified in the macrophages in the cancer phenotype. One of the up-regulated genes, *Bcl3* was found to be approximately two and a half times higher than the controls. It is a transcription co-activator proto-oncogene and a candidate for therapeutic intervention of disease progression. Bcl3 is aberrantly expressed in tumors, including breast cancer. Although the role of Bcl3 in tumor pathology is poorly understood, it mediates transcription suppression of NF-kB signaling and affects metastasis-associated genes. Bcl3 deficiency was found with only minor immunologic defects. Therefore, it may also be considered a plausible drug target [43]. Interleukin-1 receptor type 2 (IL1R2), was discovered to be a negative immune regulator, and is three-fold up-regulated in pathologic cases (Table S1). IL1R2 binds and increases the activity of the ubiquitin-specific protease 15 (USP15) in breast cancer cells. IL1R2 promotes the self-renewal of breast tumor-initiating cells, as well as cancer proliferation and invasion [44]. However, USP15 is a deubiquitinase and was found to be involved in several cellular signaling pathways such as COP9-signalosome, NFκB, and p53 signaling pathways [45]. The *in vitro* and *in vivo* study suggested that overexpression of IL1R2 leads to down-regulation of USP15, and helps in controlling breast cancer malignancy. Therefore, IL1R2 was reported to be a potential therapeutic target for breast cancer treatment [44]. Additionally, the cytokines and chemokines (Cxcl10, Cxcl9, and Cxcr3) were found to be up-regulated (Table S1) in the phenotype. These factors are suggested to play a role in driving cancer cell proliferation in the bone microenvironment [46].

Further, the extended analysis using Enrichr showed several crucial miRNAs like miR-138-5p, miR-26a-5p, miR-574-5p, hsa-miR-331-5p (Supplementary Table S4) which are targeting to DEGs. The miR-138-5p and miR-331 are associated with cell migration and invasion ability of breast cancer cells [47–49]. The miR-497 suppresses proliferation and induces apoptosis in breast cancer cells by targeting Bcl-w gene [50]. The miR-26a-5p is a well known tumor oncogene for osteosarcoma [51] and is also known to inhibit breast cancer cell growth [52]. The miR-26a-5p was found to be remarkably upregulated in osteosarcoma patients and significantly correlated with poor prognosis [53]. The miR-574-5p was found to be highly related to development and metastasis of breast cancer [54]. It was reported actively involved in the Wnt/β-catenin signaling pathway and plays crucial role in bone metastasis in non-small cell lung cancer (NSCLC) patients [55]. These miRNAs target identified DEGs which is evident from Figure 4 and Supplementary Table S4 and their interaction have been proven experimentally in literature. The miR-497 suppresses proliferation and induces apoptosis in breast cancer cells by targeting Bcl-w gene [50].

The Enrichr also showed several significant TFs (p < 0.05) which are interacting with identified DEGs (Supplementary Table S5, Figure 5A). The IRF8 (Interferon regulatory factor 8) and BATF (Basic Leucine Zipper ATF-Like Transcription Factor) are very crucial in developing the anti-tumour response in tumour surveillance. IRF and BATF negative mice had a fast rate of tumour development [56, 57]. Notably, these TFs and other TFs like STAT6 and CCDN1 are directly involved in developing breast cancer and its metastasis [58–60]. Deregulated levels of these TFs are correlated with unfavorable relapse-free survival of patients. The ROC analysis showed that Bcl3 gene possessed highest specificity and sensitivity to distinguish cancer and control samples while other identified genes failed to correlate with that (Figure 6, Supplementary Figure S1A–H, supplementary Table S6).

Selected genes were analysed for their binding to most common anti-tumour drugs (Actinomycin-D and Adrimycin) by molecular docking (Figure 7, Supplementary Table S7–S9). The Actinomycin-D (Act-D) is a well-known potent anticancer drug produced by Streptomyces bacteria. The high toxicity caused by Act-D is attributed to its ability to inhibit transcription of several important gene such as c-myc, c-met, myotonic Dystrophy type 1 etc. Importantly, it has been approved by the FDA for multiple tumors, Wilms’ tumor and gestational choriocarcinoma and in combination with other drugs to treat high risk tumor in chemotherapy regime [61–63]. The chemical structure of Act-D comprises planar phenoxazone ring and two cyclic pentapeptide lactones, a very heavy molecule [63]. The Adriamycin (synonym doxorubicin) is a glycoside antibiotic whose structure and stereochemistry are represented in Figure 6 (formula I). It consists of the tetracyclic quinoid aglycone adriamycinone (l4-hydroxydaunomycinone) linked to the aminosugar daunosamine [64, 65]. Both drugs were found to bind selected genes (ADGRG7, FABP4, IRF4) efficiently (free energy −7.6–9.4 kcal/mol; Table 1).

The study unveiled various deregulated genes demonstrating a strong role in bone metastasis of breast cancer macrophages. Distinct and altered signaling pathways were identified and it was determined that their key genes are likely involved in promoting carcinogenesis. Deregulated genes in lipid metabolism are directly involved in immunological responses, which in turn promote cancer and its spread to bone. The molecular docking study revealed that the identified deregulated genes (ADGRG-7 and IRF4) have vital anticancer drugs (Actinomycin-D and Adriamycin) targets. ROC analysis showed BCl3 gene was most specific with breast cancer-associated bone metastasis. The extended analysis by Enrichr identified miRNAs and TFs which are directly involved in the crucial pathological mechanism. However, their exact role in the pathophysiology of bone metastasis needs to be investigated within *in vitro* conditions.

## 6 Conclusions

In breast cancer bone metastasis, most immunological molecules such as chemokines, cytokines, and lipid metabolism molecules were found to be altered. These identified altered pathways promote bone metastasis outgrowth and prolonged survival of tumor cells. Some of the up-regulated genes such as Bcl3, Cxcl10, and Cxcr3; miRNAs (miR-497, miR-574, miR-138) and TFs (IRF8, STAT6, CCDN1) associated with metastasis may also be plausible targets for future therapeutics. However, *in vitro* and *in vivo* studies are required to confirm the role of these identified genes and pathways in the pathogenesis.

## Supplementary Material

Supplementary Material Details

Supplementary Material Details
