# Supplementary material for: Genome-wide expression reveals potential biomarkers in breast cancer bone metastasis
Source: J Integr Bioinform. 2022 Apr 8;19(3):20210041. doi: 10.1515/jib-2021-0041 (PMC9521824; doi:10.1515/jib-2021-0041)
Supplement: Supplementary file 1 — Supplementary Material Details [file j_jib-2021-0041_suppl_001.docx]

**Supplementary figures-**

**Fig.S1-A. ROC plot of the**  **CD209A gene**

**Fig.S1-B. ROC plot of the CD209D gene**

**Fig.S1-C. ROC plot of the FABP4 gene**

**Fig. S1-D. ROC plot of the IRF4 gene**

**Fig. S1-E. ROC plot of the LPL gene**

**Fig.S1-F. ROC plot of the MRC gene**

**Fig. S1-G. ROC plot of the VCAN gene**

**Fig. S1-H. ROC plot of the ADGRG7 gene**
